# Supplementary figures and images for: Evolutionary divergence of induced versus constitutive antiviral gene expression levels between primates and rodents
Source: PLoS Comput Biol. 2025 Jun 24;21(6):e1013165. doi: 10.1371/journal.pcbi.1013165 (PMC12187014; doi:10.1371/journal.pcbi.1013165)

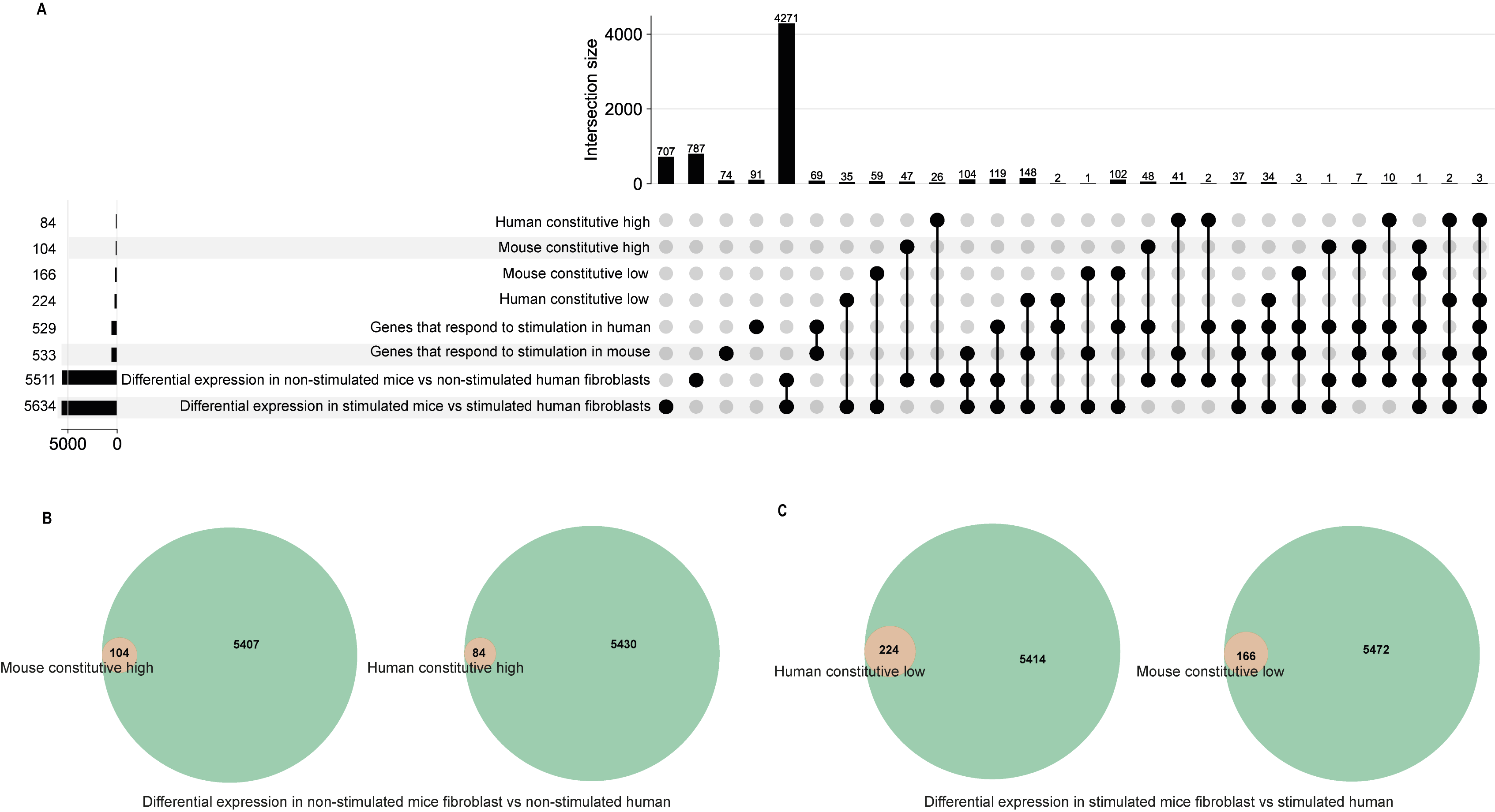

Supplement: S1 Fig — (A) An enlarged version of Fig 4C - An upset plot including the four groups of transcriptionally divergent genes in response to dsRNA between species, genes that respond to dsRNA stimulation in each species, and differentially expressed genes between human and mouse in non-stimulated conditions, and separately, in stimulated conditions. The total number of genes is shown to the left of each row, and the intersection between various groups is shown on top of each of the bars. Note that these sets show only the mutually exclusive genes, for example – the group “Genes that respond to stimulation in human” has a total of 529 genes, but most of them are part of intersections with other groups, and thus only 91 of them are shown in the fourth column. (B) Venn diagrams showing how the two groups of constitutive-high genes are part of the group of differential expression in non-stimulated mouse versus human. (C) Venn diagrams showing how the two groups of constitutive-high genes are part of the group of differential expression in non-stimulated mouse versus human. B-C demonstrate four cases of overlaps from the upset plot in A. (PNG) [file pcbi.1013165.s001.png]

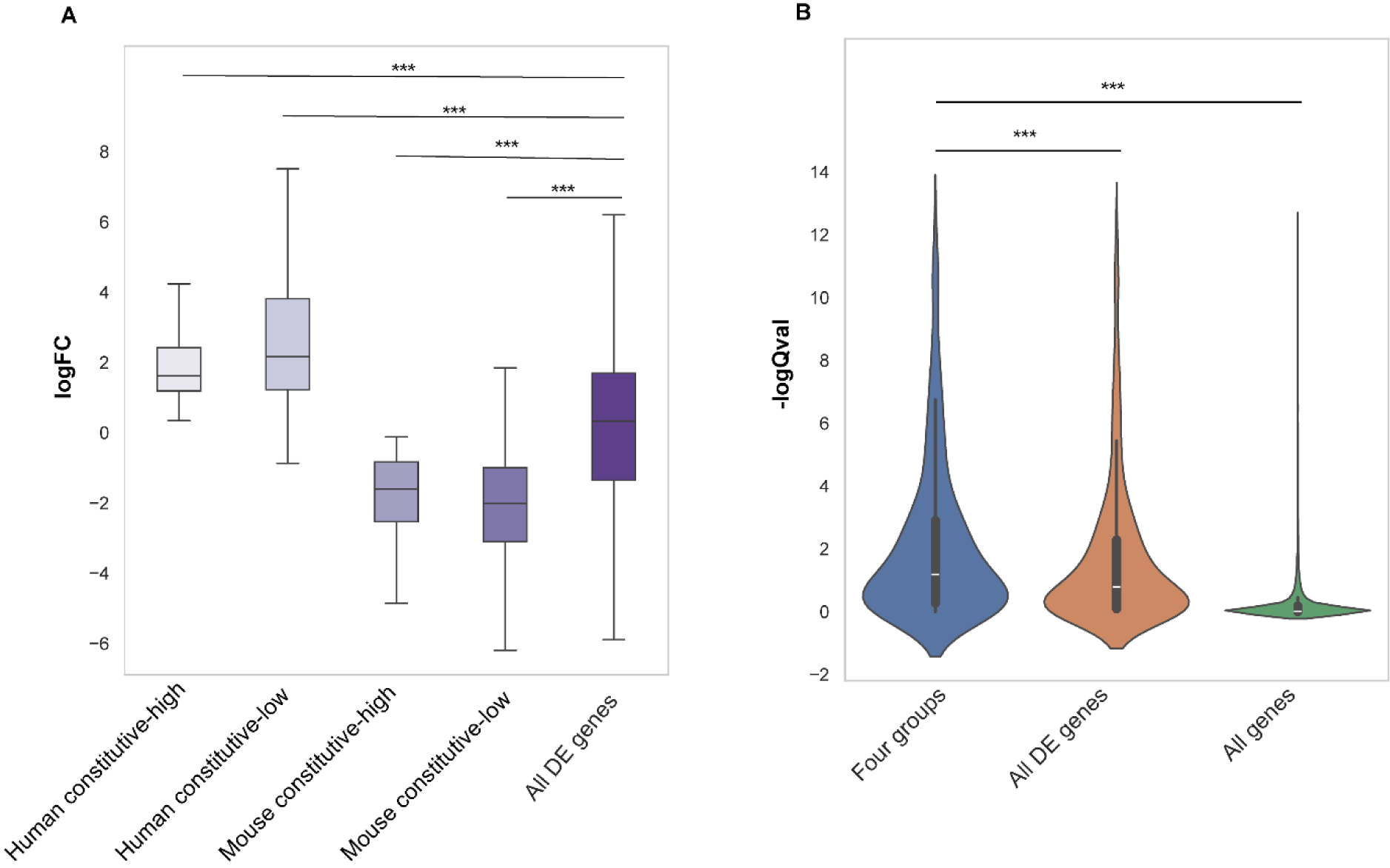

Supplement: S2 Fig — We implemented a linear model with an interaction model of species:condition. We then tested the DE values of this interaction term, of the four groups detected using the new approach described in the main text. This provides a measure to test the agreement between the new approach developed in this manuscript and a different approach to find genes that diverge in transcriptional response between species. (A) log(Fold Change) values for each of the four groups and all DE genes from the interaction term (Species:Conditions). FDR-corrected P-values are shown for one-sided Mann–Whitney test that was performed under the hypothesis that the distribution of values in human is higher and in mouse is lower than the overall DE set. (B) Q-value distribution of genes from all four groups, all DE genes, and all one-to-one human-mouse orthologs from the interaction term. FDR-corrected P-values are shown for one-sided Mann–Whitney tests. (PNG) [file pcbi.1013165.s002.png]

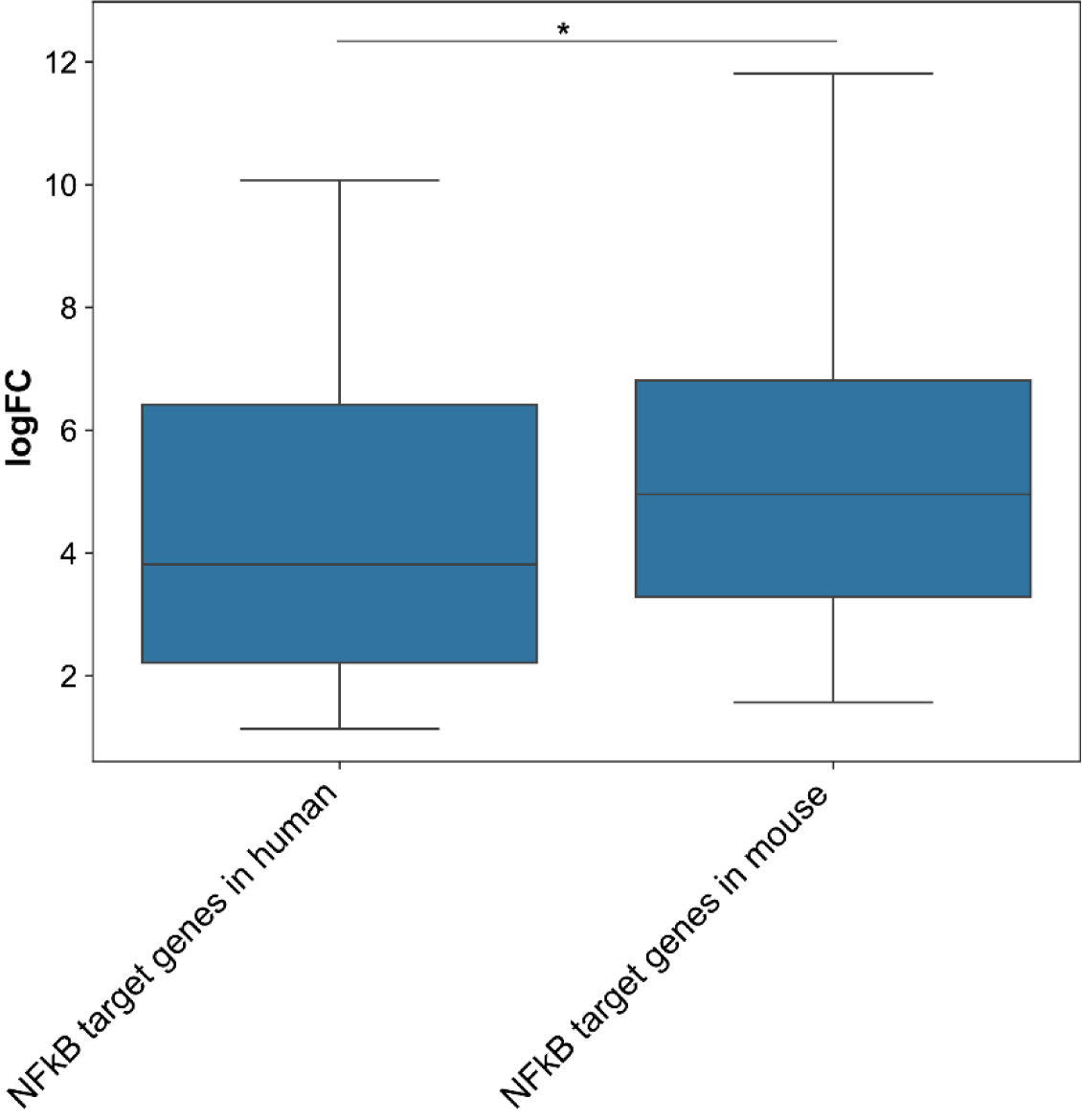

Supplement: S3 Fig — The sets were compared using a one-sided Mann-Whitney test. (PNG) [file pcbi.1013165.s003.png]

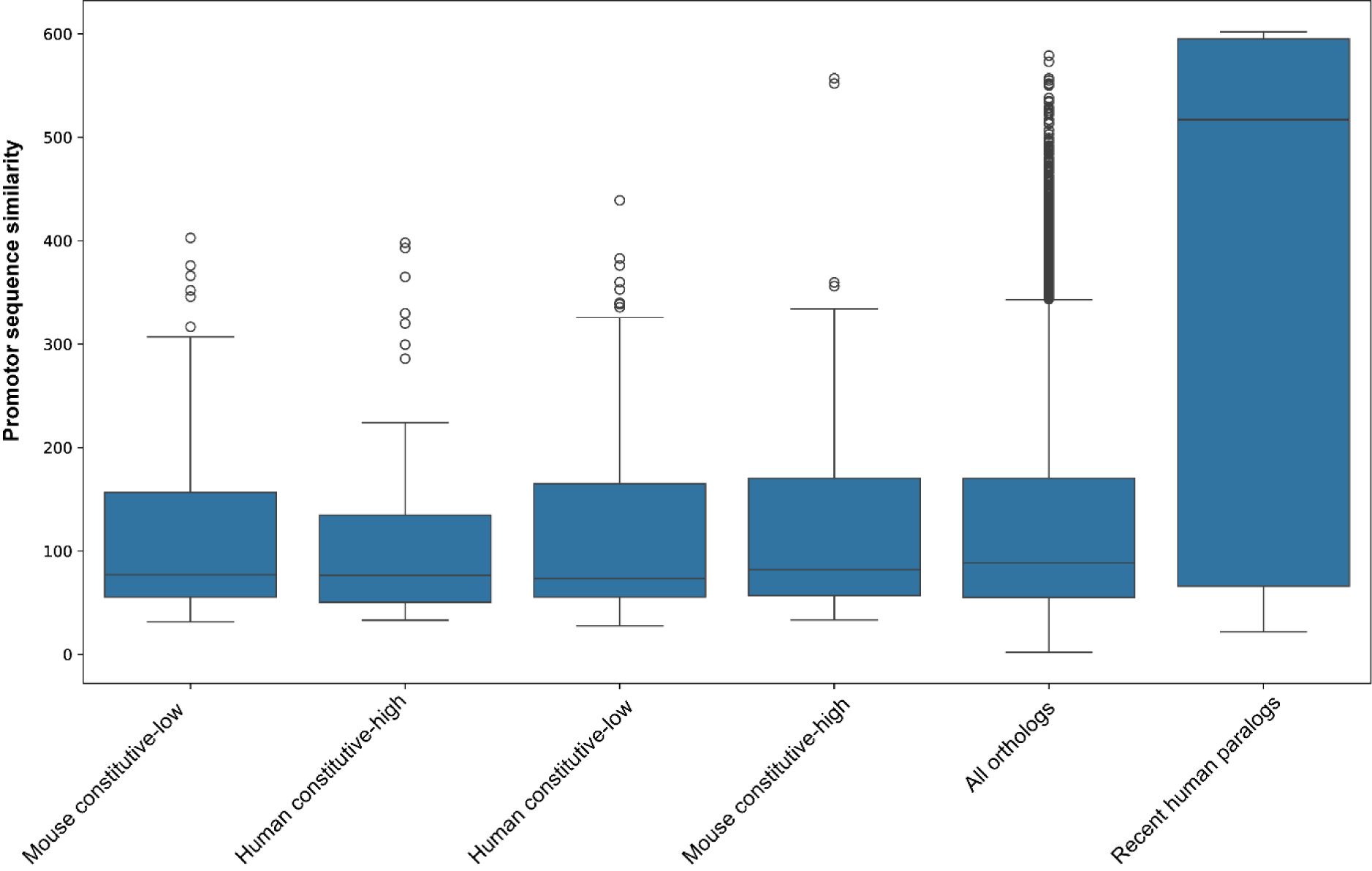

Supplement: S4 Fig — A Sequence similarity scores between promoter regions of human and mouse orthologs (the four groups defined in the manuscript and all orthologs), as well as human paralogs that have duplicated after the split between human and chimpanzee (see Fraimovitch and Hagai, BMC Biology (2023)1). This Fig demonstrates that the sequence similarity between human, and mouse orthologs in all four groups tested and in general, is very low, much lower than recent human duplicates where significant promotor sequence similarity remains. (PNG) [file pcbi.1013165.s004.png]

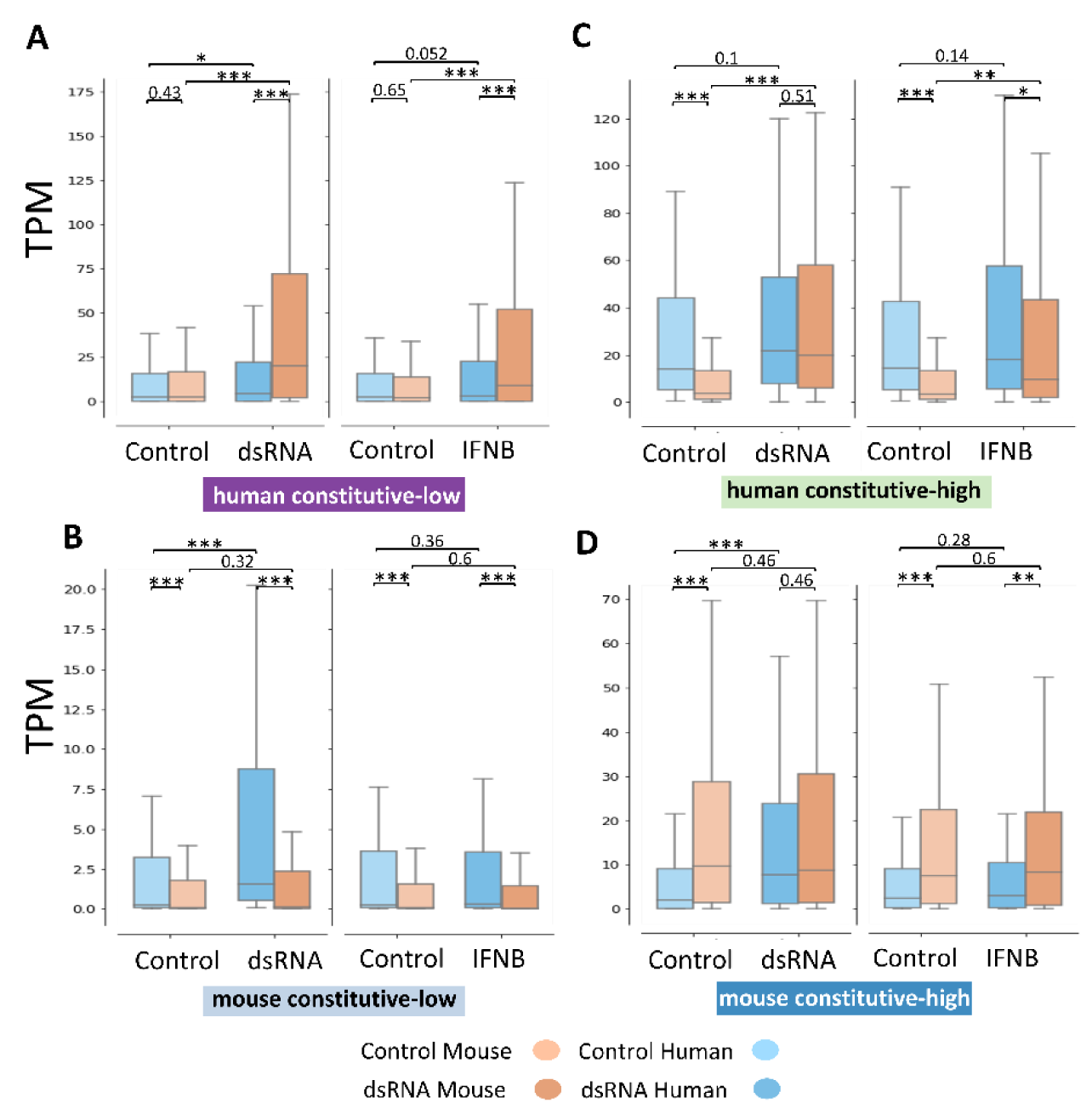

Supplement: S5 Fig — Averaged TPM levels of human and mouse genes in control and in stimulation conditions in both dsRNA and IFN systems for genes in (A) ‘human constitutive-low’, (B) mouse constitutive-low’, (C) ‘human constitutive-high’ and (D) ‘mouse constitutive-high’. FDR-corrected one- or two-sided Mann–Whitney tests were performed according to the expected pattern of expression matching to the specific group and statistical significance is shown. The observed results in A-D suggest an overall similarity in the transcriptional behavior of the human-mouse orthologs, originally identified based on their divergence in response to dsRNA, to behave similarilyy also in response to IFN. (PNG) [file pcbi.1013165.s005.png]

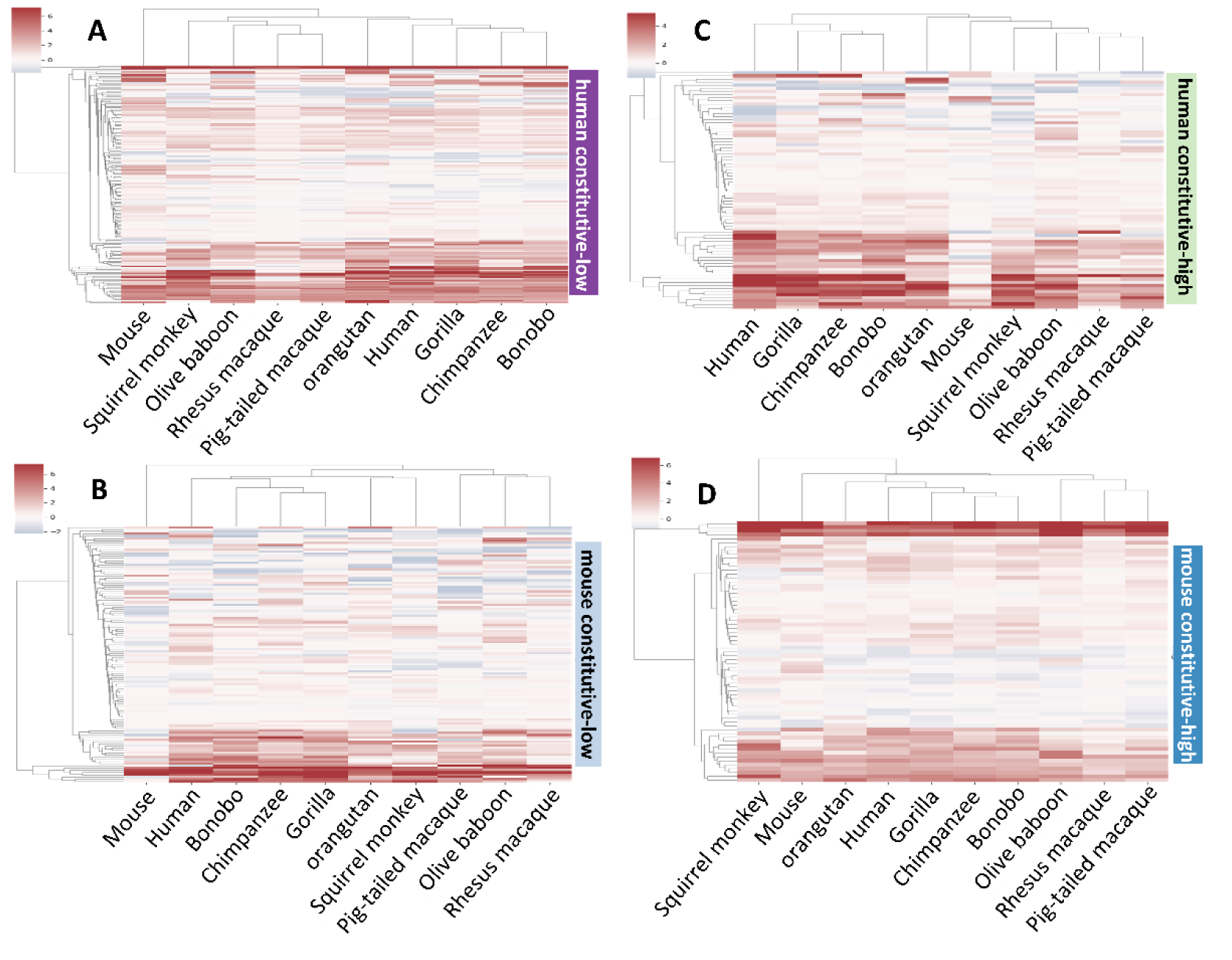

Supplement: S6 Fig — Hierarchically clustered heatmaps on logFC values in response to dsRNA for orthologous genes in cells from 9 primates and mouse, for each one of the 4 divergent gene groups, defined in Fig 3: (A) ‘human constitutive-low’, (B) mouse constitutive-low’, (C) ‘human constitutive-high’ and (D) ‘mouse constitutive-high’. The logFC values are from differential expression analysis between control and stimulation with dsRNA conditions for each of the species from the 10-species system (in each case, the DE values are within the same species). (PNG) [file pcbi.1013165.s006.png]

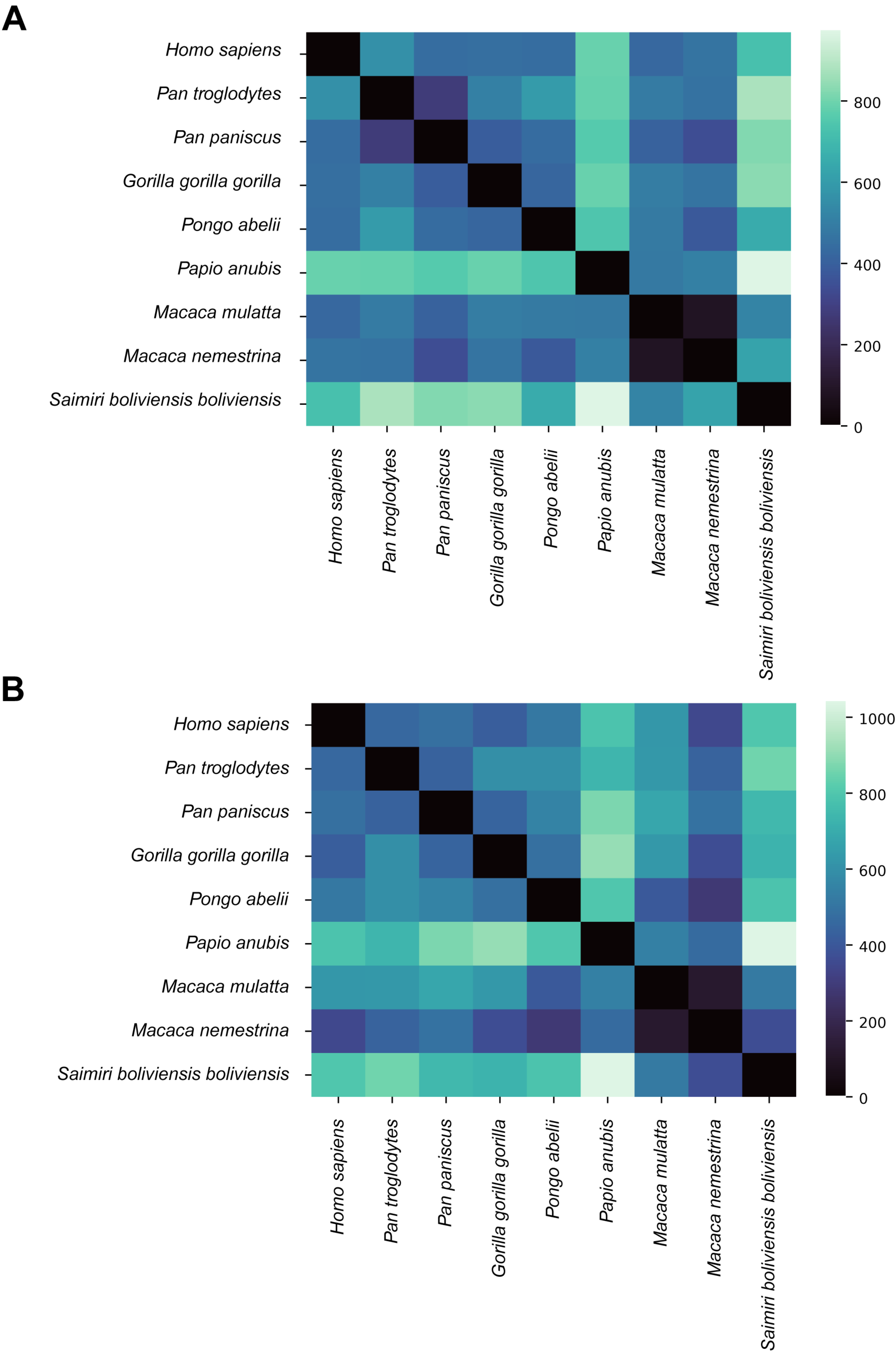

Supplement: S7 Fig — Numbers of (A) constitutive-high and (B) constitutive-low genes in each of the 9 primate pairs. Primate transcriptomics data is based on Gaska et al. [19], and the detection of the genes follows the same procedure described in the manuscript for human versus mouse data. Primates are ordered by phylogenetic distance to human. (PNG) [file pcbi.1013165.s007.png]

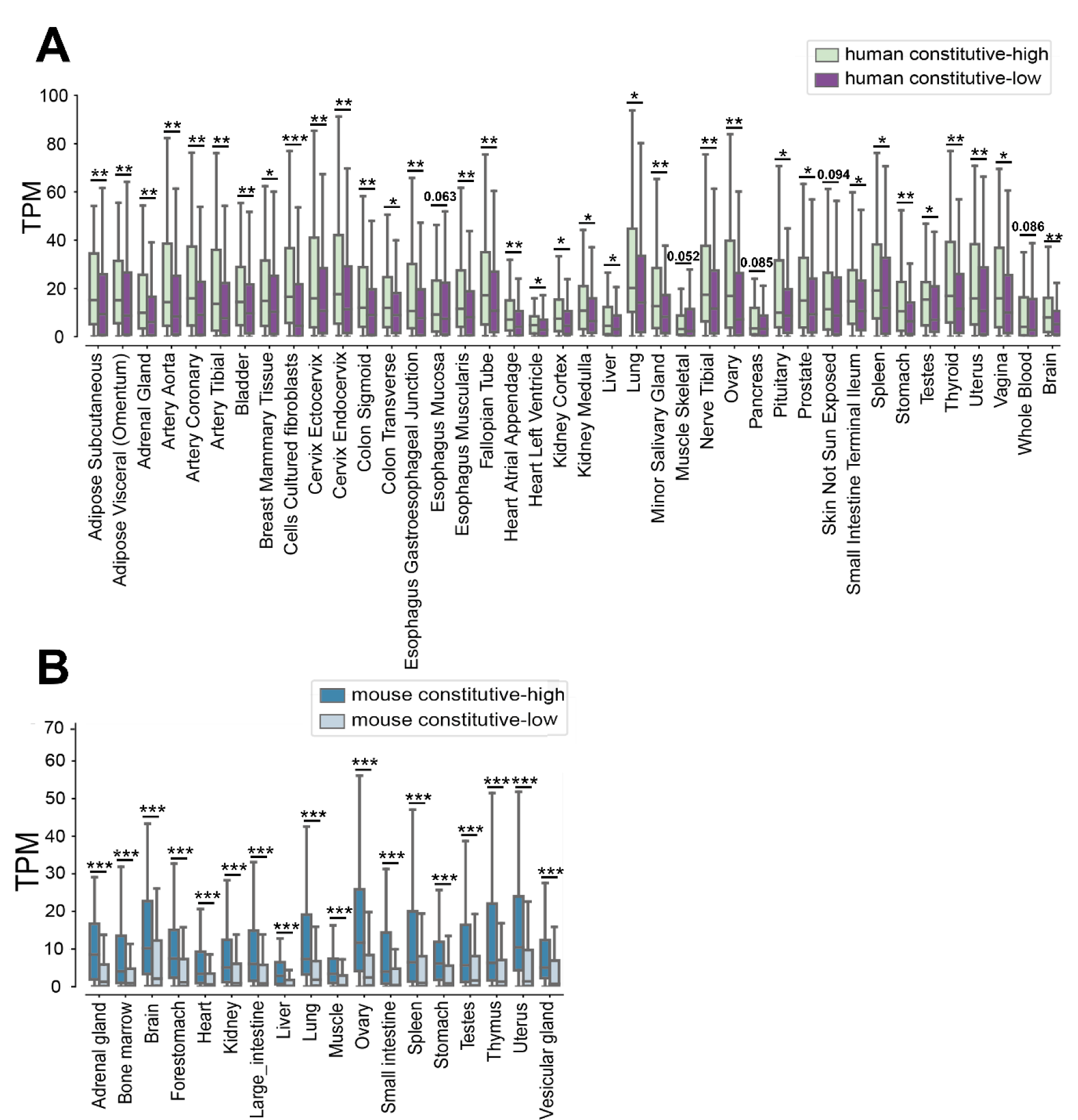

Supplement: S8 Fig — Distributions of TPM values are shown in boxplots, of constitutive-low and high genes as defined (A) for human, in 40 human tissues from the GTEx dataset [3], and (B) for mouse, in 17 mouse tissues from the BodyMap dataset [4]. FDR-corrected P-values are shown for one-sided Mann–Whitney test, performed between the basal TPM values of constitutive-high and low genes in human or mouse, for each tissue within the species. In the majority of cases (tissues/ species), the constitutive-low genes are significantly lower in expression that the constitutive-high genes. (PNG) [file pcbi.1013165.s008.png]

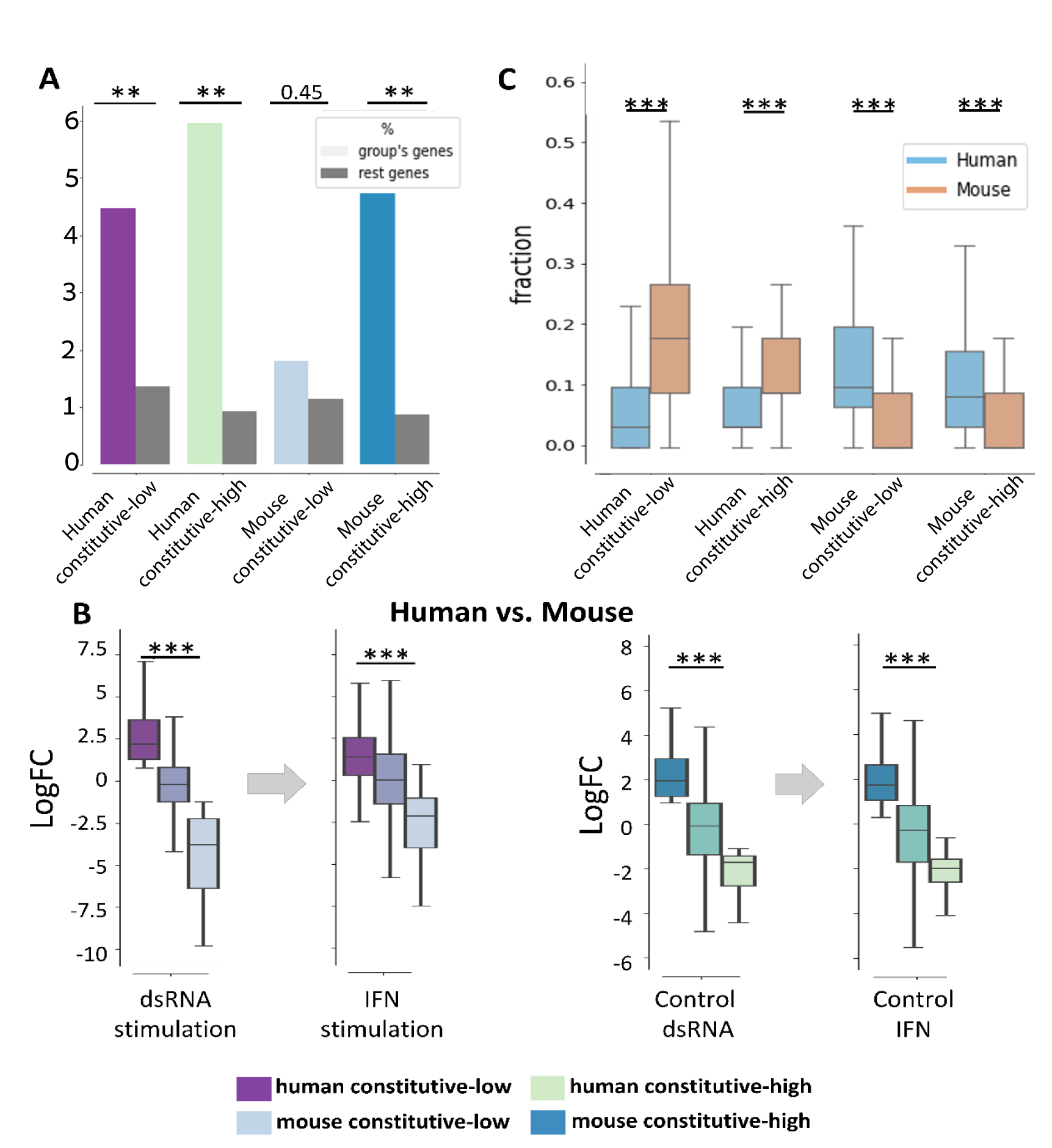

Supplement: S9 Fig — Each of the 4 divergent groups is shown, partitioned by Interferome [5] datasets of human and mouse transcriptional response data (Each dataset is based on transcriptional response to IFN in a different cell or tissue in human or mouse). FDR-corrected P-values are shown for one-sided Mann–Whitney test that was performed under the hypothesis that the fraction in human is higher (in mouse constitutive high or mouse constitutive low groups) or lower (in human constitutive high or human constitutive low groups) than in mouse. We observe that two distributions (human versus mouse) are always significantly different and that they follow our expectations based on the stimulation data from human and mouse fibroblasts. For example, in the group of genes that were identified as “human constitutive low” in human-mouse fibroblast data, we observe that the distribution of human is lower than that of mouse orthologous genes, as expected given the fact that this group is induced in mouse fibroblasts, and not in human fibroblasts, following IFN stimulation. (PNG) [file pcbi.1013165.s009.png]
